# Consequences of Prolonged Substance Use Disorder in Psychosis, ADHD and Violence: 6 Month Follow-Up Study

**DOI:** 10.3390/medsci14030377

**Published:** 2026-07-06

**Authors:** Carlos Roncero, Milton Merizalde-Torres, Diego Remón-Gallo, Lourdes Aguilar, Pilar Andrés-Olivera, Pilar González-Peláez, LLanyra García-Ullán, M. Sol Cobo, Armando González-Sánchez

**Affiliations:** 1 Department of Health Sciences, Miguel de Cervantes European University (UEMC), 47012 Valladolid, Spain; mhmerizalde@saludcastillayleon.es (M.M.-T.); mpgonzalez@uemc.es (P.G.-P.); mscobo@uemc.es (M.S.C.); 2 Instituto de Investigación Biomédica de Salamanca (IBSAL), 37007 Salamanca, Spain; d.remon@usal.es (D.R.-G.); maguilar@saludcastillayleon.es (L.A.); mpolivera@saludcastillayleon.es (P.A.-O.); mlullan@saludcastillayleon.es (L.G.-U.); armando_gonzalez@usal.es (A.G.-S.); 3 Psychiatric Unit, School of Medicine, University of Salamanca, 37007 Salamanca, Spain; 4 Network of Research In Primary Care of Addictions (RIAPAD), Instituto Carlos III, 28222 Madrid, Spain; 5 Rio Hortega Universitary Hospital, 47012 Valladolid, Spain; 6 Health Care Complex of Salamanca, 37007 Salamanca, Spain; 7 Faculty of Psychology, Pontifical University of Salamanca (UPSA), 37002 Salamanca, Spain

**Keywords:** SUD, age, psychosis, ADHD, autism, violence

## Abstract

**Background**: Substance Use Disorder (SUD) is frequently associated with psychiatric comorbidity, including psychotic symptoms, impulsivity and neurodevelopmental traits. The influence of age and duration of substance use on these clinical characteristics and on treatment retention remains insufficiently understood. **Objectives**: To examine the influence between age, duration of substance use, clinical presentation, patterns of violence, and treatment retention in individuals with SUD. **Methods**: A prospective 6-month cohort study was conducted at the Alcoholism Treatment Unit of the CAUSA Hospital Complex in Salamanca, Spain. A total of 264 patients with SUD were classified into two groups: prolonged substance use (≥55 years of age or ≥25 years of substance use; n = 127) and shorter substance use trajectories (<55 years and <25 years of substance use; n = 137). Participants completed structured clinical interviews and validated measures of quality of life, impulsivity, autistic traits, addiction severity, psychotic symptoms and violence. Non-parametric analyses were applied (α = 0.05; 95% CI). **Results**: Younger participants showed a significantly higher prevalence of auditory and visual hallucinations and persecutory delusions at baseline. During follow-up, both groups exhibited a reduction in physical aggression while driving and an increase in insults and verbal threats. No significant differences were observed in recent uncontrolled violence. Positive screening results for ADHD, autistic traits and impulsivity were not associated with treatment retention. Lower baseline physical functioning was associated with reduced completion of the 6-month follow-up assessment. **Conclusions**: Age and duration of substance use were associated with differences in the clinical presentation of SUD. Younger individuals exhibited a greater burden of psychotic symptoms and violence-related behaviours, whereas poorer physical functioning was associated with lower follow-up retention among individuals with prolonged substance use histories. These findings support the importance of age-sensitive assessment and management strategies in patients with SUD.

## 1. Introduction

According to the Diagnostic and Statistical Manual of Mental Disorders, Fifth Edition, Text Revision (DSM-5-TR), Substance Use Disorder (SUD) is characterized by a problematic pattern of substance use leading to clinically significant impairment or distress, manifested by cognitive, behavioral, and physiological symptoms [1]. SUD rarely occurs in isolation and frequently coexists with other psychiatric disorders, including psychotic disorders, Attention-Deficit/Hyperactivity Disorder (ADHD), mood disorders, and personality disorders [2,3,4]. This coexistence, currently conceptualized as Dual Disorder (DD), is associated with greater clinical severity, poorer psychosocial functioning, increased healthcare utilization, and more complex treatment needs than either condition alone [5,6,7]. Consequently, understanding the factors that influence the clinical presentation and longitudinal evolution of DD has become a major focus of contemporary addiction psychiatry [8,9].

Among the psychiatric manifestations associated with SUD, psychotic symptoms represent one of the most clinically relevant complications [9,10,11,12]. The use of substances such as cannabis [13,14,15], cocaine, and alcohol has been linked to hallucinations, delusions, and psychotic disorders, either through direct effects or by increasing vulnerability in predisposed individuals [16]. Psychotic symptoms are associated with greater addiction severity, poorer psychosocial functioning, and worse clinical outcomes [17,18,19]. However, their expression may vary according to age, gender [20] and duration of substance use [21], suggesting a dynamic interaction between addiction and psychosis throughout the lifespan [20,22,23,24].

Neurodevelopmental disorders also play an important role in the clinical heterogeneity of SUD. ADHD is highly prevalent among individuals with SUD and has been consistently associated with impulsivity, emotional dysregulation, violence, criminal behavior, and treatment difficulties [25,26,27]. Although longitudinal studies indicate that hyperactivity and impulsivity tend to decrease with age, the extent to which these changes influence the clinical course of SUD remains insufficiently understood [26,27]. Similarly, growing evidence suggests a meaningful relationship between Autism Spectrum Disorder (ASD) traits and substance use. Nevertheless, the influence of age and chronic substance exposure on autistic traits in SUD populations has received limited attention [28].

Impulsivity and violence are additional dimensions of particular relevance in DD [29]. Both have been associated with poorer clinical outcomes, greater psychosocial impairment, and increased risk of relapse. Emotional dysregulation, a common feature across ADHD, SUD, and psychotic disorders, may contribute to aggressive behaviors and interpersonal conflict [30,31,32]. Moreover, age appears to influence impulsivity and violent behavior, with younger individuals generally exhibiting higher levels of behavioral dyscontrol [28].

Age and duration of substance use are key determinants of the clinical presentation and progression of SUD, influencing physical health, psychiatric symptomatology, cognitive functioning, and treatment trajectories [33]. Previous research has independently associated psychotic symptoms, impulsivity, violence, ADHD, and ASD traits with SUD [34,35,36]; however, the interaction of these dimensions across different stages of the lifespan remains insufficiently understood. Longitudinal studies simultaneously examining these factors in routine clinical settings are scarce. Therefore, the present study aimed to compare patients with prolonged substance use trajectories with those presenting shorter exposure histories, evaluating differences in psychotic symptoms, ADHD symptoms, autistic traits, impulsivity, violence, quality of life, and treatment retention over a six-month follow-up period.

We hypothesized that younger patients and those with shorter substance use trajectories would exhibit higher levels of psychotic symptoms, impulsivity, ADHD-related symptoms, and violence, whereas patients with prolonged substance use histories would demonstrate greater physical deterioration. Additionally, we hypothesized that greater psychiatric severity, particularly psychotic symptoms, impulsivity, and neurodevelopmental traits, would be associated with lower treatment retention during follow-up.

## 2. Materials and Methods

### 2.1. Ethical Statement

Informed consent was obtained from all subjects involved in the study. The study was conducted in accordance with the Declaration of Helsinki and approved by the Ethics Committee of the Institute of Biomedicine of Salamanca (IBSAL) (approval code: PI 2022 09 1141; 1 September 2022). Appropriate measures were implemented to ensure the complete confidentiality of participants’ personal data.

### 2.2. Instruments

An initial interview was conducted to collect sociodemographic data and the patient’s medical history. SUD diagnosis was established using the Structured Clinical Interview for DSM Disorders (SCID-I) [37]. Additionally, the Addiction Severity Index (ASI/EuropASI) [38] was used to assess addiction severity and substance use-related problems, including violence-related variables. Driving-related aggressive behaviors were assessed using the Driving Aggression Test [39]. Health-related quality of life was assessed using the 36-item Short Form Survey (SF36) [40], which includes subscales for physical functioning, physical role, pain, general health, vitality, social functioning, emotional role, and mental health, with higher scores indicating better health status.

Impulsivity was assessed using the Spanish version of the Barratt Impulsiveness Scale-11 (BIS-11) [41], which evaluates cognitive, motor and non-planning impulsivity; higher scores indicate greater impulsivity. ADHD was evaluated with the Spanish version of the Adult ADHD Self-Report Scale (ASRS v1.1), a self-report tool for adult symptoms. Two scoring approaches were used: the six-item Part A screening score [42], and the full 18-item symptom score [43]. Autistic traits were measured using the Autism-Spectrum Quotient Short Version (AQ-SV) [44], a validated self-report instrument for adults without intellectual disability, also applicable to populations with SUD [44]. This 28-item assessment evaluates social skills, routines, flexibility, imagination, and attention to numerical or data details; scores ≥ 63 were considered indicative of clinically significant autistic traits [45].

The presence of psychotic symptoms was assessed using the SCID-I interview [37], which records perceptual disturbances and other psychotic symptoms [19]. Although social desirability bias is possible, the patients were informed that they would receive the treatment and support independently of the responses.

### 2.3. Procedure and Participants

A prospective cohort study was conducted involving patients with SUD undergoing standard psychopharmacological treatment. The non-probabilistic sample was recruited from a single center located in Salamanca, Spain. Baseline measurements and six-month follow-up assessments were conducted to evaluate changes in physical and psychological health in relation to substance use history. The Alcoholism Treatment Unit (UTA) at the Salamanca University Hospital Complex provides specialized care for patients referred from primary care, correctional facilities, and specialized addiction treatment services throughout the province. All patients diagnosed according to DSM-5 or DSM-5-TR with SUD who attended the UTA were invited to participate in the study. Participants were recruited between February 2020 and September 2024. At baseline, all participants underwent a comprehensive clinical assessment using structured interviews and standardized instruments. Baseline assessments were conducted at the initiation of routine clinical follow-up within the unit, provided by a multidisciplinary team comprising by psychiatrists, psychologists, nurses, and social workers. Patients were classified according to their primary substance of use; however, most participants presented polysubstance use and were analysed as a single cohort.

At six months, the assessment was repeated, excluding instruments independent of clinical change (sociodemographic questionnaires, medical history, ASI/EuropASI, AQ-Short, SCID I, and ASRS v1.1). Inclusion criteria encompassed patients diagnosed with SUD, while exclusion criteria included schizophrenia, bipolar disorder with psychotic symptoms, cognitive impairment, language barriers, or inability to attend the center. Participants were categorized into two groups based on age and duration of opioid, cocaine, alcohol, or cannabis use: the High Lifetime Consumption Group (HL), comprising individuals aged ≥55 years or with ≥25 years of use of any psychoactive substance, and the Short Lifetime Consumption Group (sL), consisting of participants < 55 years and <25 years of use. To assess therapeutic continuity, the influence of variables measured at both the initial (basal) and six-month assessments (final) on treatment retention was examined. Treatment retention was operationally defined as completion of the 6-month follow-up assessment, as verified through medical records. Participants who did not complete the second assessment were classified as dropouts.

### 2.4. Data Analysis

Group comparisons were performed using the Mann–Whitney U test for continuous variables and the χ^2^ test for categorical variables. Within-group longitudinal comparisons were conducted using the Wilcoxon signed-rank test. Odds ratios (ORs) and percentage change were calculated for violence-related outcomes. Phi coefficients were reported as measures of effect size for χ^2^ analyses involving autism traits and psychotic symptoms. Statistical significance was set at *p* < 0.05 (two-sided).

The dataset accession numbers will be provided during review.

## 3. Results

### 3.1. Sample Description

The final sample comprised 264 individuals diagnosed with SUD, including 127 participants in the HL group and 137 in the sL group. Age-related consumption patterns did not differ according to the primary substance of use: opiates (*p* = 0.123), benzodiazepines (*p* = 0.401), cannabis (*p* = 0.708), and xanthines (*p* = 0.057). In contrast, the HL group had significantly longer histories of alcohol (*p* < 0.001), cocaine (*p* = 0.019), and tobacco use (*p* < 0.001). Baseline characteristics of the study sample are summarized in Table 1.

### 3.2. General Health

After six months of follow-up, both groups showed lower SF-36 scores than at baseline (Table 2). Within-group analysis revealed a significant decline in physical functioning in the sL group (*p* = 0.043). At baseline, the HL group had significantly higher scores for general health, vitality, social functioning, and emotional role than the sL group. At the 6-month assessment, a significant decline in emotional role was observed in the sL group.

### 3.3. Violence

No significant between-group differences in violence-related outcomes were observed at baseline or at the 6-month follow-up (Table 3). Within-group analyses showed an increase in aggressive driving behaviours over time in both the HL (*p* = 0.017) and sL groups (*p* = 0.049). Comparative analysis revealed a greater overall increase in driving aggressiveness in HL (*p* = 0.006), but not in sL (*p* = 0.061).

The *p*-values of the χ^2^ test were also reported (Table 4), with the “inability to control violent behaviors in the last month” being highly significant (*p* = 0.015; OR = 5.43).

### 3.4. Impulsivity

At baseline, the sL group exhibited higher non-planning and total impulsivity scores, whereas the HL group had higher motor impulsivity scores (Table 5).

After six months, non-planning impulsivity increased in HL (*p* = 0.013), while motor impulsivity increased in sL (*p* = 0.043) (Table 6).

### 3.5. ADHD

ADHD symptoms were highly prevalent in both groups, with positive screening results in 18.3% of the HL group and 36.3% of the sL group. The HL group also demonstrated significant lower ASRS scores on both the 24-item screening, (*p* < 0.001), [HL (n = 115) mean = 9.42, SD = 5.94; sL (n = 135) mean = 11.45, SD = 5.00], and the 6-item screening (*p* < 0.001), [HL (n = 115) mean = 1.91, SD = 1.43; sL (n = 135) mean = 2.82, SD = 2.08].

### 3.6. Psychosis

Psychotic symptoms, including persecutory delusions and hallucinations, were more prevalent in the sL group than in the HL group. In contrast, no differences were found between other psychotic symptoms or the diagnosis of psychosis.

### 3.7. Factors Associated with Treatment Dropout

Higher baseline physical functioning was associated with a greater likelihood of treatment dropout in the HL group (*p* = 0.008) (Table 7). Violence-related variables, impulsivity, ADHD symptom severity, psychotic symptoms, and AQ subscale scores were not associated with treatment dropout in either group (Table 8).

## 4. Discussion

Compared with the HL group, younger participants exhibited poorer scores for general health, vitality, social functioning, and emotional role. During follow-up, both groups showed a shift from physical to verbal aggression, consistent with the behavioural de-escalation models outlined in psychotherapy interventions for SUD. Younger participants also presented a higher prevalence of psychotic symptoms and greater impulsivity than older individuals. In contrast, treatment retention appeared to be more closely associated with overall physical functioning than with violence or impulsivity, highlighting the potential value of age- and severity-specific management strategies in patients with SUD.

Compared with the HL group, the sL group showed poorer scores for general health, vitality, social functioning, and emotional role, with no significant improvement after six months of follow-up. Several explanations may account for these findings. First, the lower representation of severe cases among older participants may reflect barriers to treatment associated with physical or functional deterioration. Second, survivor bias may contribute to these results, as previous studies have shown that individuals with more severe SUD trajectories have higher mortality rates or require more intensive levels of care [46,47]. Finally, younger individuals seeking treatment for the first time may present greater clinical impairment associated with more frequent and complex substance use patterns [48]. Taken together, these findings highlight the importance of intervention strategies aimed not only at reducing substance use but also at preserving physical functioning throughout the course of the disorder.

Younger individuals exhibited greater difficulty controlling violent behaviours. One of the main findings of this study was that, following treatment, both groups showed a reduction in physical aggression accompanied by an increase in verbal aggression. Although previous studies have reported inconsistent findings, there is evidence that treatment is more effective in reducing physical than verbal violence [49,50]. One possible explanation is that physical aggression is more closely related to impulsivity and emotional dysregulation, whereas verbal aggression may reflect more deliberate forms of interpersonal communication [51]. The reduction in physical aggression may therefore be associated with improvements in emotional regulation during treatment, while the increase in verbal aggression could represent a displacement from physical to verbal expression. Consequently, verbal aggression may require additional therapeutic approaches, particularly cognitive-behavioural interventions targeting anger management and interpersonal communication [52].

A possible explanation is that psychological interventions may partially compensate for neurobiological alterations associated with long-term substance use. Chronic exposure to psychoactive substances has been linked to dysfunction in monoaminergic systems involved in emotional regulation and impulse control [52,53]. Although the present study did not assess neurobiological markers, the reduction in physical aggression suggests that treatment may improve behavioral regulation even when underlying neurobiological vulnerabilities persist. Thus, psychological interventions targeting emotional regulation may remain beneficial across different stages of addiction.

In contrast to previous studies reporting lower treatment adherence among patients with greater violence and clinical instability [53], the most violent individuals in our cohort were more likely to remain in treatment. Although the mechanisms underlying this finding remain uncertain, violence may represent a marker of greater clinical severity and reinforce the need to incorporate anger management and violence-focused interventions into SUD treatment. At baseline, younger participants exhibited greater difficulties in decision-making and higher overall impulsivity, whereas older individuals or those with prolonged substance use histories showed higher motor impulsivity. Previous studies have associated elevated impulsivity with poorer treatment adherence [54]; therefore, early retention strategies may be particularly important for patients with marked impulsive traits, given the well-established benefits of sustained treatment engagement on long-term outcomes [55,56]. Furthermore, impulsivity appeared to remain elevated among participants with ADHD who continued treatment, particularly in the HL group. This finding suggests that prolonged substance use may be associated with persistent ADHD symptomatology, highlighting the need for targeted interventions addressing impulsivity in this population.

In the HL group, the reduction in impulsivity and hyperactivity suggests that these symptoms may decline with age. Among adolescent samples, impulsivity has been characterized by a preference for immediate, lower-value rewards over larger, delayed rewards [57]. This, in the long term, may influence the initiation and persistence of substance use. Although impulsivity has been extensively investigated in adolescents and young adults, evidence in older individuals with SUD remains limited. Several mechanisms may explain these findings. First, longitudinal studies have shown that hyperactive-impulsive symptoms of ADHD tend to decrease with age [57,58]. Second, survivor bias may contribute to this pattern, as individuals with greater impulsivity and more severe comorbidities, including ADHD and SUD, have a reduced life expectancy [20,59]. Indeed, previous longitudinal studies have reported increased mortality among individuals with the hyperactive-impulsive presentation of ADHD, particularly from causes related to impulsive behaviours [26]. Finally, age-related differences in the psychometric properties of the ASRS have also been described [58]. Taken together, these findings suggest that the relationship between age, impulsivity, and SUD is multifactorial and should be interpreted within a lifespan perspective.

A notable finding was the high prevalence of ASD traits among individuals with SUD, affecting 45.7% of the HL group and 54.3% of the sL group, with no significant between-group differences. ASD traits may influence substance use patterns, treatment response, and the management of violent behaviours, particularly among younger individuals. One possible explanation is the self-medication hypothesis [60], whereby substance use serves as a coping strategy for social and emotional difficulties. These findings support the need to adapt SUD treatment to the specific needs of this population [61]. Differences between groups were observed only for the imagination subscale, which reflects cognitive flexibility and mental simulation abilities. This finding may be related to age-associated reductions in cognitive flexibility [54] and/or the neurotoxic effects of prolonged substance use on brain regions involved in executive functioning, including the hippocampus, prefrontal cortex, and frontotemporal networks [62,63]. Although ASD traits were not associated with treatment retention, their presence may complicate the clinical management of SUD because of their frequent overlap with ADHD and psychotic symptoms [64].

The present study confirmed the high prevalence of psychotic symptoms among individuals with SUD, consistent with previous reports [18,65]. However, psychotic symptoms were less prevalent among older participants. This difference may be related to the higher prevalence of cocaine use in the younger group, as cocaine has been consistently associated with an increased risk of psychotic episodes, and because the peak incidence of psychosis typically occurs during the second and third decades of life [66]. Younger participants also exhibited higher levels of impulsivity, supporting previous evidence of an association between impulsivity and psychotic symptoms in individuals with [18] and without SUD [67]. Although earlier studies linked psychotic symptoms and impulsivity to violent behaviour among stimulant users [68], no such association was observed in the present cohort. This discrepancy may reflect the absence of methamphetamine users, the substance most strongly associated with violence in previous reports. Nevertheless, the early identification and management of psychotic symptoms remain clinically relevant, given their association with greater addiction severity [69].

No consistent pattern of factors associated with treatment retention was identified, consistent with previous studies reporting that treatment retention is not consistently predicted by violence, impulsivity, psychotic symptoms, or ADHD [70,71]. Survivor bias should also be considered when interpreting these findings, as individuals with long-standing dual disorders and greater functional impairment are more likely to require residential or long-term care, thereby reducing their representation in outpatient cohorts [72]. This interpretation is further supported by the elevated mortality rates reported among individuals with SUD and psychotic disorders, resulting from both medical causes and suicide [73,74,75]. Consequently, the apparent reduction in the prevalence of some clinical conditions over time may reflect not only genuine clinical improvement but also the selective loss of participants with poorer health, who are more likely to discontinue treatment, miss follow-up assessments, transition to higher-intensity services, relocate, or die prematurely. As a result, patients remaining in outpatient follow-up may represent a relatively more functional subgroup despite persisting impulsivity, interpersonal conflict, or psychiatric symptoms, including psychotic symptoms and ASD traits. This mechanism may also partly explain the lower clinical severity observed among older participants.

The present study has several limitations. First, the use of a non-probabilistic sample and recruitment from a single outpatient center may limit the generalizability of the findings to other SUD populations. Second, given the high prevalence of polysubstance use, years of use were analyzed separately for each substance rather than as integrated patterns of combined use, which may have provided a more comprehensive characterization of clinical profiles [76]. Third, attrition during follow-up reduced the sample available for longitudinal analyses, a common challenge in SUD research [77,78]. Treatment retention was operationalized as completion of the 6-month follow-up assessment; however, because reasons for missing follow-up evaluations were not systematically recorded, non-retention may reflect different clinical or administrative circumstances. Additionally, the detailed dropout-management discussion and sensitivity analyses suggested by the reviewer were considered beyond the scope of the manuscript and therefore are not presented in the article, although they were performed internally for review. Finally, neurobiological measures, such as neuroimaging, were not included, and individuals with schizophrenia or bipolar disorder with psychotic features were excluded. Despite these limitations, the study was conducted in a homogeneous outpatient clinical sample, supporting the applicability of the findings to routine clinical practice.

In conclusion, younger individuals with SUD exhibited a higher prevalence of psychotic symptoms, greater impulsivity, and more violence-related behaviours than older participants or those with prolonged substance use histories. The observed shift from physical to verbal aggression during follow-up highlights the potential value of psychological interventions targeting emotional regulation and interpersonal functioning. However, it is also possible that discontinuing use may temporarily exacerbate symptoms, as the potential self-medication effect diminishes. The high prevalence of ADHD- and ASD-related traits further supports the importance of routinely assessing neurodevelopmental characteristics in patients with SUD. Although several clinical differences were observed according to age and duration of substance use, the mechanisms underlying these associations remain uncertain. Given the observational design of the study, these findings should be interpreted as associations rather than causal relationships. Overall, the results support the implementation of age-sensitive assessment and treatment strategies that prioritize treatment retention, early identification of psychotic symptoms and neurodevelopmental traits, and interventions tailored to the clinical profile of each patient.

Future studies should include larger, multicenter cohorts and incorporate neurobiological measures, such as neuroimaging, to further investigate the mechanisms underlying the associations observed in this study. In addition, larger samples would allow comparisons of longitudinal outcomes across specific substance use disorder subgroups, thereby improving the understanding of how different patterns of substance use influence clinical presentation, treatment retention, and long-term outcomes.

## 5. Conclusions

In this cohort of patients with Substance Use Disorder, younger individuals exhibited higher levels of violence, impulsivity, and psychotic symptoms, whereas older patients or those with prolonged substance use histories showed greater functional deterioration. The observed shift from physical to verbal aggression during follow-up highlights the potential relevance of emotional regulation strategies in clinical practice. High rates of ADHD- and ASD-related traits were also identified across the sample, supporting the importance of routinely assessing neurodevelopmental features in SUD populations. Although several clinical differences were observed according to age and duration of substance use, the mechanisms underlying these associations remain unclear. Overall, these findings suggest that the clinical presentation of SUD may vary across different stages of life and support the development of age-sensitive assessment and treatment approaches. Further longitudinal and multicenter studies are needed to clarify the relationships among psychiatric symptoms, neurodevelopmental traits, violence, and treatment outcomes.

## Figures and Tables

**Table 1:** Sociodemographic characteristics of the sample.

Characteristic	HL	sL	Total
**Sex (sample size)**	127	137	264
Male	95	95	190
Female	32	42	74
**Age (mean, SD, range: min.–max.)**	59.37(6.73) 41–80	41.54 (8.32) 22–54	50.12 (11.71) 22–80
**Civil Status**			
Single	20	57	77
Married/Coupled	44	53	97
Divorced/Separated	26	24	50
Widowed	8	2	10
Lost data	29	1	30
**Education level**			
Cannot read or write	0	2	2
Incomplete primary education	4	9	15
School certificate	39	50	89
High school diploma or vocational training	36	45	81
University education	19	31	50
Lost data	29	0	27
**Nationality**			
Spaniards	94	124	218
European (excluding Spaniards)	2	4	6
Africa	0	1	1
Latin America	1	8	9
Lost data	30	0	30
**Etnia**			
European	96	125	221
African descent	1	6	7
East Asian descent	1	0	1
Others	0	6	6
Lost data	29	0	29
**Years of consumption (mean, SD, range: min.–max.)**			
Opioids	0.62 (2.83) 0–20	0.12 (1.00) 0–11	0.33 (1.98) 0–20
Cocaine	3.18 (7.434) 0–38	3.12 (5.38) 0–23	3.14 (6.28) 0–38
Alcohol	19.85 (15.43) 0–65	8.36 (6.63) 0–24	13.05 (12.43) 0–65
Cannabis	5.39 (11.31) 0–44	3.53 (6.42) 0–24	4.29 (8.78) 0–44
Benzodiazepines	4.77 (8.67) 0–40	2.60 (4.63) 0–25	3.49 (6.65) 0–40
Tobacco	31.82 (15.06) 0–56	19.66 (11.77) 0–42	24.63 (14.48) 0–56

SD: Standard Deviation. HL: High Lifetime Consumption Group. sL: Short Lifetime Consumption Group.

**Table 2:** Changes in clinical variables from baseline to follow-up considering groups.

SF-36	HL			sL			*p*-Value for Comparison Between Groups	
	Basal (n = 118) Mean (SD)	Final (n = 28) Mean (SD)	*p*-Value	Basal (n = 136) Mean (SD)	Final (n = 27) Mean (SD)	*p*-Value	Basal	Final
Physical Functioning	74.32 (28.98)	58.0 (30.51)	0.361	82.98 (20.40)	68.9 (28.81)	0.043 *	0.206	0.166
Role-Physical	44.69 (42.58)	31.6 (36.77)	0.687	38.6 (42.48)	34.0 (37.84)	0.672	0.453	0.745
Bodily Pain	58.08 (30.28)	41.4 (37.62)	0.270	55.78 (28.90)	45.0 (29.31)	0.451	0.074	0.453
General Health	54.28 (20.86)	44.1 (26.80)	0.387	49.48 (22.54)	45.5 (24.73)	0.626	0.038 *	0.601
Vitality	50.84 (19.7)	39.1 (25.72)	0.241	45.96 (18.89)	44.2 (22.64)	0.752	0.011 *	0.582
Social Functioning	63.78 (30.13)	53.4 (42.67)	0.569	53.49 (32.08)	52.3 (30.85)	0.741	<0.001 **	0.766
Role-Emotional	46.91 (43.10)	48.1 (46.21)	0.736	27.20 (39.37)	19.9 (32.82)	0.176	0.004 **	0.008 **
Mental Health	53.87 (20.19)	46.9 (28.39)	0.572	46.59 (18.56)	46.5 (22.22)	0.883	0.206	0.886

Mann–Whitney U test was performed. * *p* < 0.05; ** *p* < 0.01. SD: Standard Deviation. HL: High Lifetime Consumption Group. sL: Short Lifetime Consumption Group.

**Table 3:** Comparison of the groups in the initial and final measurements and influence of the treatment on the aggressiveness test while driving.

Subscale of Aggression While Driving	Basal						Final						Basal–Follow-Up			
	HL		sL		*p*-Value	OR–PC	HL		sL		*p*-Value	OR–PC	HL		sL	
	Yes	No	Yes	No			Yes	No	Yes	No			*p*-Value	OR–PC	*p*-Value	OR–PC
Insults	8	69	12	68	0.386	0.657–34.3%	8	19	9	19	0.840	0.888–11.1%	0.017 *	2.851–185.1%	0.049 *	0.373–62.7%
Threats	3	74	9	71	0.083	0.319–68.0%	4	23	3	25	0.648	1.449–44.9%	0.051	1.056–5.63%	0.938	0.946–5.4%
Vehicles	1	76	2	78	0.583	0.513–48.7%	0	27	2	26	0.157	NC	NC	NC	0.263	NC
People	0	77	1	79	0.325	NC	0	27	1	27	0.322	NC	NC	NC	0.433	NC
Total	12	296	24	296	0.052	0.500–50.0%	12	96	15	97	0.683	0.808–19.1%	0.006 **	0.233–76.6%	0.061	0.524–47.5%

χ^2^ test and Odds Ratio with percentage of change (PC) were performed. * *p* < 0.05; ** *p* < 0.01. HL: High Lifetime Consumption Group. sL: Short Lifetime Consumption Group.

**Table 4:** Initial assessment of violence using EuropASI.

Studied Behavior	HL		sL		*p*-Value	OR–PC
	Yes	No	Yes	No		
Violent offenses throughout lifetime	12	76	20	96	0.483	0.757–24.2%
Inability to control violent behavior throughout lifetime	24	64	45	71	0.085	0.591–40.8%
Inability to control violent behaviors in the past month	2	86	13	103	0.015 *	0.184–81.6%

χ^2^ test and Odds Ratio with percentage of change (PC) were performed; * *p* < 0.05.

**Table 5:** Total score and cognitive, motor, and non-planning impulsivity subscales of the BIS-11.

	Impulsivity Type	HL (n = 116)	sL (n = 136)	*p*-Value
Basal	Cognitive	19.41 (3.478)	20.41 (3.296)	0.075
	Motor	21.48 (3.620)	20.70 (4.496)	<0.001 **
	Non-planning	25.52 (4.264)	27.48 (3.887)	0.033 *
	Total	66.41 (8.477)	68.59 (8.158)	<0.001 **
		**(n = 27)**	**(n = 27)**	
Final	Cognitive	19.52 (3.468)	20.22 (3.191)	0.277
	Motor	20.93 (5.284)	22.89 (5.591)	0.646
	Non-planning	27.11 (4.163)	26.59 (4.254)	0.077
	Total	64.78 (15.950)	69.70 (8.866)	0.456

Means and standard deviations for each subscale by group are presented. * *p* < 0.05; ** *p* < 0.01. SD: Standard Deviation. HL: High Lifetime Consumption Group. sL: Short Lifetime Consumption Group.

**Table 6:** Pre and post-treatment impulsivity comparison using the Wilcoxon test by group.

Impulsivity Type	HL			sL		
	Pre (n = 116)	Post (n = 27)	*p*-Value	Pre (n = 136)	Post (n = 27)	*p*-Value
Cognitive	19.61 (3.46)	19.41 (3.47)	0.473	20.67 (3.77)	20.41 (3.29)	0.875
Motor	20.87 (4.60)	21.48 (3.62)	0.345	23.13 (5.21)	20.70 (4.49)	0.043 *
Non planning	26.35 (4.33)	25.52 (4.26)	0.013 *	27.82 (5.14)	27.48 (3.88)	0.441
Total	65.92 (11.41)	66.41 (8.47)	0.580	71.52 (11.30)	68.59 (8.15)	0.465

Means and standard deviations (in parentheses) for each subscale by group. * *p* < 0.05. SD: Standard Deviation. HL: High Lifetime Consumption Group. sL: Short Lifetime Consumption Group.

**Table 7:** Factors associated with treatment dropout.

Variable	Subscale	HL			sL		
		Dropout	Full	*p*-Value	Dropout	Full	*p*-Value
Health status	Physical functioning	78.85 (23.42)	65.37 (27.92)	0.008 **	83.30 (20.79)	81.67 (19.11)	0.400
	Role limitations	49.43 (43.30)	33.26 (40.33)	0.068	38.75 (42.01)	37.96 (45.13)	0.825
	Bodily pain	61.15 (28.76)	53.67 (33.01)	0.348	57.29 (28.70)	49.67 (29.46)	0.247
	General health	55.99 (20.52)	52.00 (21.44)	0.486	50.50 (22.62)	45.37 (22.19)	0.346
	Vitality	53.10 (18.06)	46.07 (23.51)	0.290	45.73 (19.26)	46.85 (17.66)	0.782
	Social functioning	67.10 (29.29)	54.19 (32.82)	0.120	52.75 (33.52)	56.48 (25.80)	0.642
	Role emotional	45.35 (42.08)	57.64 (46.00)	0.264	25.99 (38.31)	32.11 (43.85)	0.490
	Mental health	54.74 (19.02)	52.56 (24.77)	0.879	46.39 (18.34)	47.41 (19.81)	0.974
Violence type	Insults	6	2	0.756	9	3	1
	Threats	3	0	0.366	7	2	0.838
	Property damage	1	0	0.606	2	0	0.408
	Physical injury	0	0	NC	1	0	0.561
Impulsivity	Cognitive	19.64 (3.48)	19.52 (3.46)	0.976	20.89 (3.90)	20.22 (3.19)	0.45
	Motor	20.85 (4.41)	20.93 (5.28)	0.718	23.19 (5.14)	22.89 (5.59)	0.774
	Non planning	26.12 (4.37)	27.11 (4.16)	0.437	28.13 (5.31)	26.59 (4.25)	0.188
	Total	66.27 (9.71)	64.78 (15.95)	0.804	71.97 (11.82)	69.70 (8.86)	0.264
Autism	Social behaviour	52.47 (11.17)	51.08 (6.95)	0.841	54.11 (6.10)	49.20 (8.54)	0.097
	Social skills	14.60 (4.79)	14.04 (3.21)	0.933	17.17 (5.32)	14.19 (4.33)	0.166
	Routines	10.87 (2.20)	9.80 (2.15)	0.259	9.78 (2.10)	10.13 (2.08)	0.666
	(in) Flexibility	9.27 (2.76)	8.46 (2.06)	0.356	10.11 (2.89)	8.39 (2.77)	0.073
	Imagination	17.73 (3.54)	18.40 (4.66)	0.537	17.06 (1.89)	16.28 (3.67)	0.74
	Numbers and data	11.33 (3.85)	11.08 (3.48)	0.777	13.11 (3.99)	12.23 (4.02)	0.167
	Total Score	63.80 (11.11)	62.16 (6.57)	0.738	66.78 (8.47)	60.63 (9.65)	0.067
ASRS v.1.1	A Section	1.88 (1.437)	2.04 (1.455)	0.568	2.77 (2.143)	3.04 (1.829)	0.388
	B Section	8.89 (3.635)	11.23 (10.505)	0.502	11.24 (5.088)	12.30 (4.648)	0.313

Sample sizes (n) varied by instrument: Health status: HL Dropout (n = 87), HL Full (n = 27); sL Dropout (n = 109), sL Full (n = 27). Violence type: HL Dropout (n = 61), HL Full (n = 16); sL Dropout (n = 60), sL Full (n = 20). Impulsivity: HL Dropout (n = 89), HL Full (n = 27); sL Dropout (n = 109), sL Full (n = 27). Autism (AQ): HL Dropout (n = 62), HL Full (n = 16); sL Dropout (n = 70), sL Full (n = 17). Autism (ASRS): HL Dropout (n = 89), HL Full (n = 26); sL Dropout (n = 108), sL Full (n = 27). At violence type, the values indicate frequencies; zero values indicate that, although data were collected, there was an absence of that type of violence. NC: Not Calculated. HL: High Lifetime Consumption Group. sL: Short Lifetime Consumption Group. ** *p* < 0.01.

**Table 8:** Comparison between dropouts and psychosis presence.

Variable	HL					sL				
	Full		Dropout		*p*-Value	Full		Dropout		*p*-Value
	Yes	No	Yes	No		Yes	No	Yes	No	
Psychotic symptoms	5	21	14	55	0.908	8	19	40	68	0.472
Persecutory delusions	1	25	4	65	0.704	2	24	18	89	0.243
Hallucinations	1	25	5	64	0.544	3	24	25	82	0.162
Auditory hallucinations	1	25	3	66	0.914	1	26	17	90	0.097
Visual hallucinations	0	26	6	63	0.120	2	25	16	91	0.304
Kinesthetic hallucinations	0	26	2	67	0.380	0	27	1	105	0.774
Other hallucinations	0	26	2	67	0.380	0	27	0	107	NC
Psychotic disorder	1	25	1	69	0.461	0	27	7	102	0.176

NC: Not calculated. HL: High Lifetime Consumption Group. sL: Short Lifetime Consumption Group.

## Data Availability

The original contributions presented in this study are included in the article. Further inquiries can be directed to the corresponding author.

## Abbreviations

The following abbreviations are used in this manuscript:

DD	Dual disorder
SUD	Substance Use Disorder
AUD	Alcohol Use Disorder
CUD	Cannabis Use Disorder
ADHD	Attention deficit hyperactivity disorder
ASD	Autism Spectrum Disorders
SCID-I	Structured Clinical Interview for DSM Disorders
ASI	Addiction Severity Index
SF36	36-item Short Form Survey
BIS-11	Barratt Impulsiveness Scale-11
ASRS	Adult ADHD Self-Report Scale
AQ-SV	Autism-Spectrum Quotient Short Version
HL	High Lifetime Consumption Group
sL	Short Lifetime Consumption Group
OR	Odds ratio
